# Effects of the Oral Administration of Viable and Heat-Killed *Streptococcus bovis* HC5 Cells to Pre-Sensitized BALB/c Mice

**DOI:** 10.1371/journal.pone.0048313

**Published:** 2012-10-29

**Authors:** Aline D. Paiva, Kenner M. Fernandes, Roberto S. Dias, Alípio S. Rocha, Leandro L. de Oliveira, Clóvis A. Neves, Sérgio O. de Paula, Hilário C. Mantovani

**Affiliations:** 1 Departamento de Microbiologia, Universidade Federal de Viçosa, Viçosa, Minas Gerais, Brazil; 2 Departamento de Biologia Geral, Universidade Federal de Viçosa, Viçosa, Minas Gerais, Brazil; Federal University of Pelotas, Brazil

## Abstract

Antimicrobial peptides have been suggested as an alternative to classical antibiotics in livestock production and bacteriocin-producing bacteria could be added to animal feeds to deliver bacteriocins in the gastrointestinal (GI) tract of ruminant and monogastric animals. In this study, viable (V) and heat-killed (HK) *Streptococcus bovis* HC5 cells were orally administered to pre-sensitized mice in order to assess the effects of a bacteriocin-producing bacteria on histological parameters and the immune response of the GI tract of monogastric animals. The administration of V and HK *S. bovis* HC5 cells during 58 days to BALB/c mice did not affect weight gain, but an increase in gut permeability was detected in animals receiving the HK cells. Viable and heat killed cells caused similar morphological alterations in the GI tract of the animals, but the most prominent effects were detected in the small intestine. The oral administration of *S. bovis* HC5 also influenced cytokine production in the small intestine, and the immune-mediated activity differed between V and HK cells. The relative expression of IL-12 and INF-γ was significantly higher in the small intestine of mice treated with V cells, while an increase in IL-5, IL-13 and TNF-α expression was only detected in mice treated with HK cells. Considering that even under a condition of severe challenge (pre-sensitization followed by daily exposure to the same bacterial immunogen) the general health of the animals was maintained, it appears that oral administration of *S. bovis* HC5 cells could be a useful route to deliver bacteriocin in the GI tract of livestock animals.

## Introduction

Lactic acid bacteria (LAB) represent one of the best studied groups of microorganisms, and many species are known to produce antimicrobial substances. Besides their antimicrobial activity, several strains of LAB are also able to enhance innate and adaptive immunity, a feature that has been demonstrated in animal models [1], [2] and human trials [3], [4], [5].

*Streptococcus* is a genus of diverse gram-positive lactic acid bacteria. Some species of Streptococci have occasionally been associated with pathogenicity, and implicated in diseases as sore throat, pneumonia, meningitis, endocarditis, necrotizing fasciitis, ruminal acidosis and bloat [6]. On the other hand, many strains are non-pathogenic and occur as natural commensal bacteria in different habitats, including the skin, the oral cavity and the respiratory, gastrointestinal, and urogenital tracts of humans and animals [6].

*Streptococcus bovis*, a member of the group D of Lancefield [7], is an indigenous gastrointestinal Streptococci in both humans and animals [8]. In ruminants, *S. bovis* is normally present at populations ranging from 10^4^ to 10^7^ colony forming units (CFU)/g of ruminal contents [9], but it can outgrow other ruminal bacteria under optimal growth conditions [10], [11]. When cattle are fed high grain diets, *S. bovis* can produce large amounts of lactic acid and cause ruminal acidosis, a common ruminal disorder in feedlots [12].

*Streptococcus bovis* HC5 was isolated from the bovine rumen [13], and this bacterium produces the type A lantibiotic bovicin HC5, a bacteriocin active against several Gram-positive bacteria isolated from milk, silage, soil and the bovine rumen, including species of *Streptococcus*, *Clostridium*, *Listeria*, *Bacillus*, *Lactococcus*, *Alicyclobacillus* and *Staphylococcus* [14], [15], [16]. Previous work indicated that bovicin HC5 is mostly cell-associated and it can be transferred directly to sensitive bacteria [17], [18]. *S*. *bovis* HC5 can inhibit many commensal and pathogenic organisms [13], [19], and it appears that it could be used as a vehicle for bacteriocin delivery in the GI tract of livestock animals [16], [18], [19].

Since their discovery in the first half of the 19^th^ century, antibiotics have been extensively used in livestock production as therapeutic agents and growth promoters. In many countries, antibiotic therapy is still the first choice to combat microbial infections in livestock animals, and their efficacy and cost-effectiveness contribute to their popularity. Nevertheless, the continuous use of antibiotics has led to the emergence of multidrug-resistant microbial strains that no longer respond to antibiotic therapy [20], as well as antibiotic residue in animal products [21].

A number of strategies that do not depend on antibiotics have been proposed to control microbial pathogens and to improve growth and feed efficiency in livestock animals. The potential alternatives include bacteriocins, probiotic microorganisms and bacteriophages [22], [23], [24].

The application of bacteriocins to livestock has been mostly achieved by feeding bacteriocin-producing bacteria to the animals [23]. Because cell-associated bovicin HC5 is more active and stable than the cell-free bacteriocin [17], [25], *S*. *bovis* HC5 could be directly applied to manipulate feedstuffs digestion and prevent microbial infections in ruminant and monogastric animals.

Previous work demonstrated that immunization by parenteral route of livestock animals could induce strong mucosal antibody responses after challenging with bacterial cells [26], [27]. Although the humoral response against live and dead *S. bovis* cells has been demonstrated in ruminant animals [26], the cellular immune response and the direct effect of *S. bovis* on the intestinal mucosa of livestock is much less understood, particularly in monogastric animals. Therefore, the aim of the present study was to investigate the effects of the oral administration of *S*. *bovis* HC5 cells on the integrity of the gastrointestinal tract and on cytokine production in BALB/c mice that were previously sensitized with *S. bovis* cells.

Subcutaneous injections were used to overcome the possible oral tolerance of BALB/c mice to *S. bovis* HC5 cells, by priming mice to develop a more prominent *S. bovis* HC5 immune response. Our results indicated that under a condition of severe challenge, the daily administration of *S. bovis* HC5 cells resulted in histological and morphometric alterations in the intestine, and a distinguishable immunostimulatory response was elicited by viable and heat-killed *S. bovis* HC5 cells.

## Materials and Methods

### *Streptococcus bovis* HC5 and growth conditions

*Streptococcus bovis* HC5 was cultivated under anaerobic conditions, at 39°C, in basal medium containing (per liter): 0.292 g K_2_HPO_4_; 0.292 g KH_2_PO_4_; 0.48 g (NH_4_)_2_SO_4_; 0.48 g NaCl; 0.1 g MgSO_4_.7H_2_O; 0.064 g CaCl_2_.2H_2_O; 0.5 g cystein hydrochloride; 4 g Na_2_CO_3_; 0.1 g trypticase; 0.5 g yeast extract. Glucose was added as carbon source (4 g l^−1^).

The cells were grown until mid-exponential-phase (10^9^ CFU ml^−1^ per OD unit), harvested by centrifugation (1,742×*g*, 15 min, 5°C), and then washed twice with sterile phosphate buffer saline (PBS, 10 mM phosphate buffer, 150 mM NaCl, pH 7.2). Heat-killed *S*. *bovis* HC5 were prepared by autoclaving (121°C, 20 min) mid-exponential-phase cultures (prepared as described above). Previous work demonstrated that bovicin HC5 is highly heat stable and it remains associated to the producer cells even after autoclaving (13). After treatment, *S. bovis* HC5 cells were resuspended in PBS (10 mM, pH 7.2), and stored at −20°C until use.

### Ethics Statement

The BALB/c mice used in this study were housed in an animal facility at the Universidade Federal de Viçosa, according to standards and guidelines as set forth in the Animal Welfare Legislation, the Guide for the Care and Use of Laboratory Animals, the Association for the Assessment and Accreditation of Laboratory Animal Care (AAALAC) and the National Council for Animal Experimentation Control (CONCEA), following approval by the Institutional Animal Care and Use Committee (IACUC) of the Universidade Federal de Viçosa under the protocol number CEUA/UFV 97/2011.

### Experimental animals

Five-week-old female BALB/c mice weighing 18.0±1.0 g were obtained from the animal facilities of the Universidade Federal de Viçosa. Because previous studies indicate that immune responses of the gut mucosal immune system differ when live or dead cells are administered to animal models (26,27), the BALB/c mice were randomly divided into three experimental groups, containing 4 animals per group: Group 1, untreated mice (negative control, NC group); Group 2, mice given viable cells of *S. bovis* HC5 (V group); Group 3, mice given heat-killed cells of *S. bovis* HC5 (HK group). Two independent experiments were performed and a total number of eight animals were used per experimental group.

### Sensitization and administration of *S. bovis* HC5 to BALB/c mice

Considering the fact that tolerogenic immune responses of the gut mucosal immune system are often generated via oral administration of antigens, the BALB/c mice were initially sensitized in order to overcome oral tolerance. Animals belonging to the V and HK groups were subcutaneously sensitized at day zero with viable or heat-killed streptococci cells (100 µl of a 1×10^10^ CFU ml^−1^ cell suspension), respectively, using aluminum hydroxide (Al(OH)_3_) as adjuvant (50 µl of a 20 mg ml^−1^ solution in sterile saline buffer). After three weeks (day 21), each mice group were subcutaneously boosted (without the use of aluminum hydroxide) with viable or heat-killed streptococci cells. In the negative control group, sterile PBS (10 mM, pH 7.2) was administered to the animals using the same procedure described above (Al(OH)_3_ administration at day 0 and without adjuvant at day 21).

Viable or heat-killed cells (100 µl of a 1×10^10^ CFU ml^−1^ cell suspension) or PBS (100 µl) were orally administered by daily gavages to the V, HK and NC groups, respectively, without the use of adjuvant and using *18-gauge* stainless steel feeding needles. The oral administration started one week after the second sensitization (day 28) and continued during the 30-day experimental period. The mice were weekly weighted and monitored daily for the general appearance and development of adverse reactions.

### Gut permeability

The gut permeability was determined by the uptake of β-lactoglobulin (β-LG) following challenge, as described by Knippels *et al*. [28], with some modifications. At the end of the trial period (day 58), animals sensitized with *S. bovis* HC5 (viable or heat-killed cells) and negative control animals were orally challenged with 200 µl of the respective samples [*S. bovis* HC5 (viable and heat-killed cells) or PBS] to improve the chance of detecting an increase in gut permeability. After thirty minutes, the animals received an oral dose (0.2 ml of a 100 mg ml^−1^ solution in distilled water) of β-lactoglobulin (Sigma Chemicals Co., St. Louis, MO, 90% of purity).

Blood samples were collected from the orbital plexus under light isoflurane anesthesia after 0.5, 1, 2 and 5 h of the β-LG administration. The samples were kept at room temperature for 2 hours, and the sera were centrifuged at 12,000×*g* for 5 min (Eppendorf®, Centrifuge 5415C, Hamburg, Germany), at room temperature. Sera were used for the quantification of β-LG by Fast Protein Liquid Chromatography (FPLC), using a cationic exchange column (Mono Q HR 5/5).The column was equilibrated with buffer A (20 mM Tris) and the β-LG was eluted using a linear gradient of 25 to 50% buffer B (20 mM Tris, 1 M NaCl), 22°C, and at a flow rate of 1 ml min^−1^.The absorbance was monitored at 220 and 280 nm.

In order to determine the concentration of β-LG in animal sera, a calibration curve was prepared, using solutions with known β-LG concentrations (0; 6.25; 12.5; 25.0; 50.0 mg ml^−1^), mixed to pre-immune serum of the animals from each group. The pre-immune serum corresponded to the sera collected from the animals prior to the initial sensitization procedure. The pre-immune serum was also collected from the orbital plexus under light isoflurane anesthesia. The standard curve was constructed by plotting the peak areas related to β-lactoglobulin against the concentration of the standard solutions used. Serum samples before β-LG administration were used as negative control. Analyses were performed in duplicate and the chromatographic profiles obtained were compared to the calibration curve, in order to determine the β-LG levels in serum samples.

### Histological and morphometric analysis

The heart, liver, spleen and gut of all the mice were aseptically collected at the end of the experiment (day 58). The organs were washed in PBS buffer (10 mM, pH 7.2), fixed in Carson formalin solution [29], dehydrated and embedded in resin (Historesin®, Leica). Transverse and longitudinal, 3 µm thick tissue sections were obtained and stained with hematoxylin and eosin (H&E) or with Alcian Blue (pH 2.5) combined with periodic acid-Schiff (PAS) [30], depending on the histological analysis to be performed.

To each animal, ten fields of longitudinal sections stained with H&E were randomly selected and visualized with a 10× objective lens in order to perform the morphological analysis of the organs selected (specifically in the gut, villi height and villi width were determined in an area of 17 mm^2^ per animal, and for mucosal thickness the average of twenty measurements was obtained to each animal). The spleen cells were counted using ten fields of longitudinal sections visualized with a 40× objective lens, in an area of 0.23 mm^2^ per animal. For quantitative and qualitative analysis of goblet cells, ten fields of longitudinal sections (area of 1 mm^2^) stained with Alcian Blue-PAS were randomly selected and visualized with a 20× objective lens; the mucins produced by goblet cells were identified by differential staining (acid mucins in blue (AB^+^ cells), neutral mucins in red (PAS^+^ cells), and mixed acid and neutral mucins in purple (PAS/AB^+^ cells)).

Digital images of the slides were captured with the light microscope Olympus AX 60, coupled to a digital camera (Q-Color 3, Olympus). The morphometric analyzes were performed with the image analysis program Image Pro Plus 4.0 for Windows (Media Cybernetics). The results were shown as mean value±standard error of the mean.

### Analysis of relative gene expression by real-time reverse transcription (RT)-PCR

The whole spleen and jejunum segments (100 mg of tissue) were aseptically removed from all the animals within each experimental group (N = 8), washed in sterile PBS (10 mM, pH 7.2), and individually manipulated. Cells obtained from the spleen were washed with saline (0.85%), centrifuged at 7,500×*g* for 5 min (Eppendorf®, Centrifuge 5415C, Hamburg, Germany), at room temperature, and the erythrocytes were lysed using a hemolytic solution (155 mM NH_4_Cl, 10 mM KHCO_3_, pH 7.2). The splenocytes were centrifuged again and the supernatant was discarded. A jejunum segment of 6 cm was removed and washed three times with saline (0.85%), for removal of waste.

The splenocytes and jejunum segments were homogenized in Tri Reagent (Sigma®) to isolate total RNA, following the manufacturer's instructions. RNA yield were analyzed spectrophotometrically at 260 nm (Ultraspec 3000, Pharmacia Biotech, Piscataway, NJ). From each group (NC, V and HK) three samples from different animals showing highest RNA yield (≥1.5 µg.µl^−1^) were chosen for cDNA synthesis. Complementary DNA (cDNA) was synthesized through a RT reaction (M-MuLV reverse transcriptase, Promega), according to the manufacturer's instructions. Real-time RT-PCR was conducted in triplicate with a final volume of 25 µL containing 2.5 ng of cDNA, SYBR-green PCR Master Mix (Applied Biosystems, Warrington, UK), oligo(dT) cDNA as the PCR template, and 450 nM specific primers. Real-time RT-PCR was performed on the Gene Amp®5700 Sequence Detection System Version 1.3 (Applied Biosystems) and the cycling parameters used were 95°C for 10 min, 40 cycles at 94°C for 1 min, 56°C for 1 min, and 72°C for 2 min, followed by the standard denaturation curve. Primers used to amplify the murine cytokine genes (Table 1) have been previously described by Cardoso *et al.* [31].

**Table 1:** Sequences of sense (S) and antisense (AS) *primers* used for real time-PCR analysis.

*Primers*	Sequences
β-actin S	5′ AGC TGC GTT TTA CAC CCT TT 3′
β-actin AS	5′ AAG CCA TGC CAA TGT TGT CT 3′
IL-10 S	5′ TGG ACA ACA TAC TGC TAA CC 3′
IL-10 AS	5′ GGA TCA TTT CCG ATA AGG CT 3′
IL-4 S	5′ CTG ACG GCA CAG AGC TAT TGA 3′
IL-4 AS	5′ TAT GCG AAG CAC CTT GGA AGC 3′
IL-5 S	5′ GAG GTT ACA GAC ATG CAC CAT T 3′
IL-5 AS	5′ TCA GTT GGT AAC ATG CAC AAA G 3′
IL-13 S	5′ ACC AAC ATC TCC AAT TGC AA 3′
IL-13 AS	5′ ATG CAA TAT CCT CTG GGT CC 3′
TNF-α S	5′ TGT GCT CAG AGC TTT CAA CAA 3′
TNF-α AS	5′ CTT GAT GGT GGT GCA TGA GA 3′
IL-12 p40 S	5′ AGC ACC AGC TTC TTC ATC AGG 3′
IL-12 p40 AS	5′ GCG CTG GAT TCG AAC AAA G 3′
IFN-γ S	5′ GCA TCT TGG CTT TGC AGC T 3′
IFN-γ AS	5′ CCT TTT TCG CCT TGC TGT TG 3′
TGF-β S	5′ GCT GAA CCA AGG AGA CGG AAT 3′
TGF-β AS	5′ GCT GAT CCC GTT GAT TTC CA 3′
IL-17 S	5′ GCT CCA GAA GGC CCT CAG A 3′
IL-17 AS	5′ CTT TCC CTC CGC ATT GAC A 3′

The results were demonstrated as relative level of gene expression in the experimental group, by reference to the β-actin gene in each sample, using the cycle threshold (Ct) method. Measurements were conducted in duplicate and the fold increase expression was calculated by using the expression 2?DCt, according to the instructions from Applied Biosystems User's Bulletin #2 (P/N 4303859). Results were shown as mean values±standard error of the mean.

### Statistical analysis

The results were evaluated by analysis of variance (ANOVA) followed by different post tests: Dunn's multiple comparison or t test. A probability value of less than 0.05 was considered statistically significant, and all the comparisons were performed using the GraphPad Prism 5.00 software (GraphPad Software, San Diego California, USA).

## Results

Daily oral administration of viable and heat-killed *S. bovis* HC5 cells to pre-sensitized BALB/c mice started at day 28 and continued daily uninterruptedly for 30 days. All mice survived during the experimental period and differences in the general appearance, behavior and adverse reactions were not observed.

### The weight gain of BALB/c mice is not affected by *S. bovis* HC5

The weight of BALB/c mice belonging to the three experimental groups, negative control (NC), mice given viable *S. bovis* HC5 cells (V), mice given heat-killed *S. bovis* HC5 cells (HK), was monitored during the trial period in order to verify if the sensitization followed by challenge with *S. bovis* HC5 could induce weight loss in the animals, which is a clinical manifestation frequently associated to gastrointestinal disorders.

**Figure 1:**
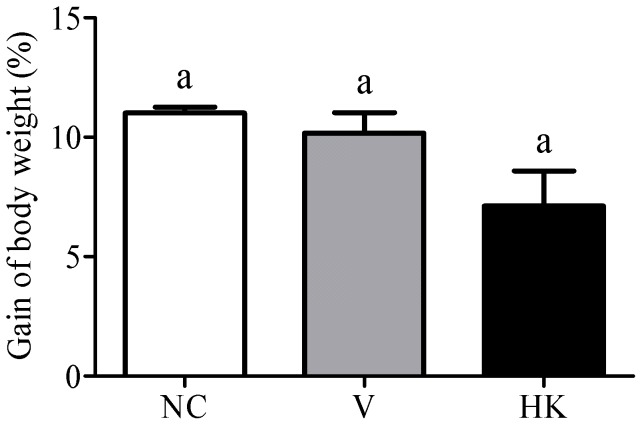
Effect of the oral administration of *S. bovis* HC5 on percent weight gain of BALB/c mice. The weight of the animals is visualized as percentage of the animals' weight, which was calculated comparing the weight at the end of the experiment (day 58) to the weight at the day of the first sensitization (day 0). Each bar represents the mean value from six determinations with the standard error of the mean (SEM). Treatments with identical lowercase letters were considered not statistically significant by the Dunn's multiple comparison test (p>0.05). (NC) negative control group; (V) mice treated with viable *S. bovis* HC5 cells; (HK) mice treated with heat-killed *S. bovis* HC5 cells.

Prior to the beginning of the experiment, no significant differences were detected among the average weight of the mice (18.51, 18.0 and 18.18 g to NC, V and HK groups, respectively). Among the mice of the NC group, the average weight ranged from 18.51±0.35 g (day 0) to 20.79±0.31 g (day 58), which means a weight gain of 11.01% along the trial period. After 58 days of experiment, the average weight of the animals from the V and HK groups (20.03, 19.60 g, respectively) was lower compared to the NC group (20.79 g) (p<0.05). However, the percentage of weight gain did not differ among the NC, V and HK groups (11.01%, 10.2% and 7.24%, respectively (p>0.05)) (Figure 1).

### Gastrointestinal permeability is altered upon oral administration of heat-killed *S. bovis* HC5 cells

In order to evaluate possible changes in gut permeability induced by *S. bovis* HC5, a β-LG uptake assay was performed. β-LG was easily identified by the FPLC method developed in this study, and the retention time of β-LG was 10.68 min. It was possible to observe a clear increase of peak areas, which was proportional to the increase of the β-LG concentrations in the animal sera (y = 0.0238x−0.0417; R^2^ = 0.9912).

**Figure 2:**
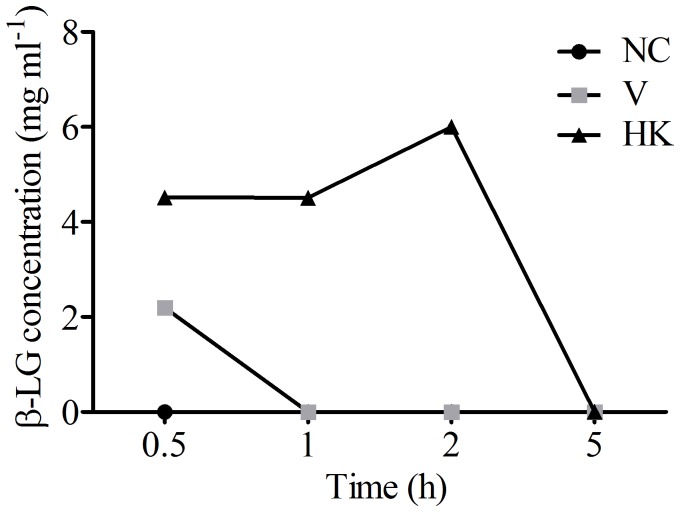
β-lactoglobulin levels in animal sera from the treatment groups. An intragastrically dose of β-LG (20 mg) was administered as a bystander protein to the negative control group and the groups that received *S. bovis* HC5 (viable and heat-killed cells). At the indicated time points following β-LG administration, the levels of β-LG in mice sera were determined by FPLC. The results show an average of the β-LG level detected in a pool of animal's sera from each group. β-LG was not detected in all serum samples from negative control group. (NC) negative control group; (V) mice treated with viable *S. bovis* HC5 cells; (HK) mice treated with heat-killed *S. bovis* HC5 cells (HK).

In V group, increased uptake of β-LG was detected only in sera obtained 0.5 h after the β-LG administration (2 mg ml^−1^). In sera obtained from animals of the HK group, a significant amount of β-LG was detected at 0.5, 1 and 2 h after β-LG administration (4.52 mg ml^−1^, 4.51 mg ml^−1^ and 6.0 mg ml^−1^, respectively). After 5 h of administration, the clearance of β-LG was completed and β-LG could not be detected in the sera of the animals from all tested groups. No β-LG was detected in serum samples obtained before β-LG administration or in samples from negative control animals after administration of β-LG (Figure 2).

### Histological and morphometric changes are observed in spleen and gut of BALB/c mice after *S. bovis* HC5 administration

*S. bovis* HC5 has not been associated with infection and disease, but some *S. bovis* strains are known to cause clinical symptoms in livestock animals and humans [8], [32]. Therefore, we decided to investigate histological and morphometric alterations caused by *S. bovis* HC5 in some organs of the BALB/c mice used in this study.

**Figure 3:**
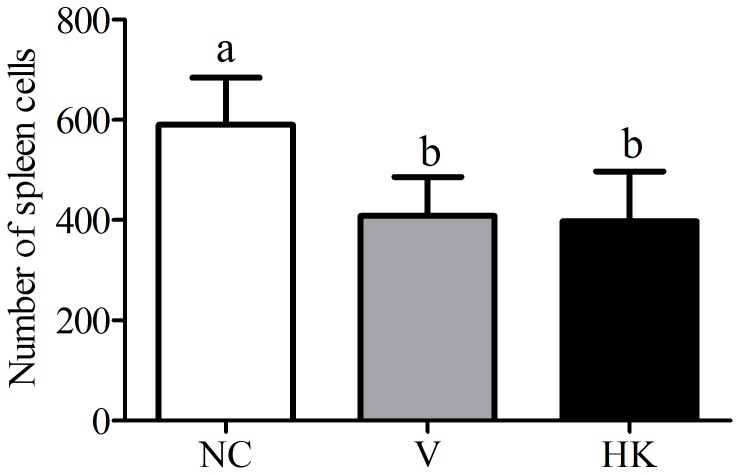
Number of total spleen cells among the experimental groups. Data represent the average±SEM. Differences among treatments were indicated by different lowercase letters and were considered statistically significant by the Dunn's multiple comparison test (p<0.05). (NC) negative control group; (V) mice treated with viable *S. bovis* HC5 cells; (HK) mice treated with heat-killed *S. bovis* HC5 cells.

No alterations were identified in the heart of the animals in all the groups evaluated. Also the livers showed a preserved lobular architecture and cellularity of parenchyma, without cellular infiltrates or parenchymal substitutions (data not shown). However, a significant decrease in the total number of spleen cells in the animals from the V and HK groups was observed, when compared to the NC group (Figure 3).

**Figure 4:**
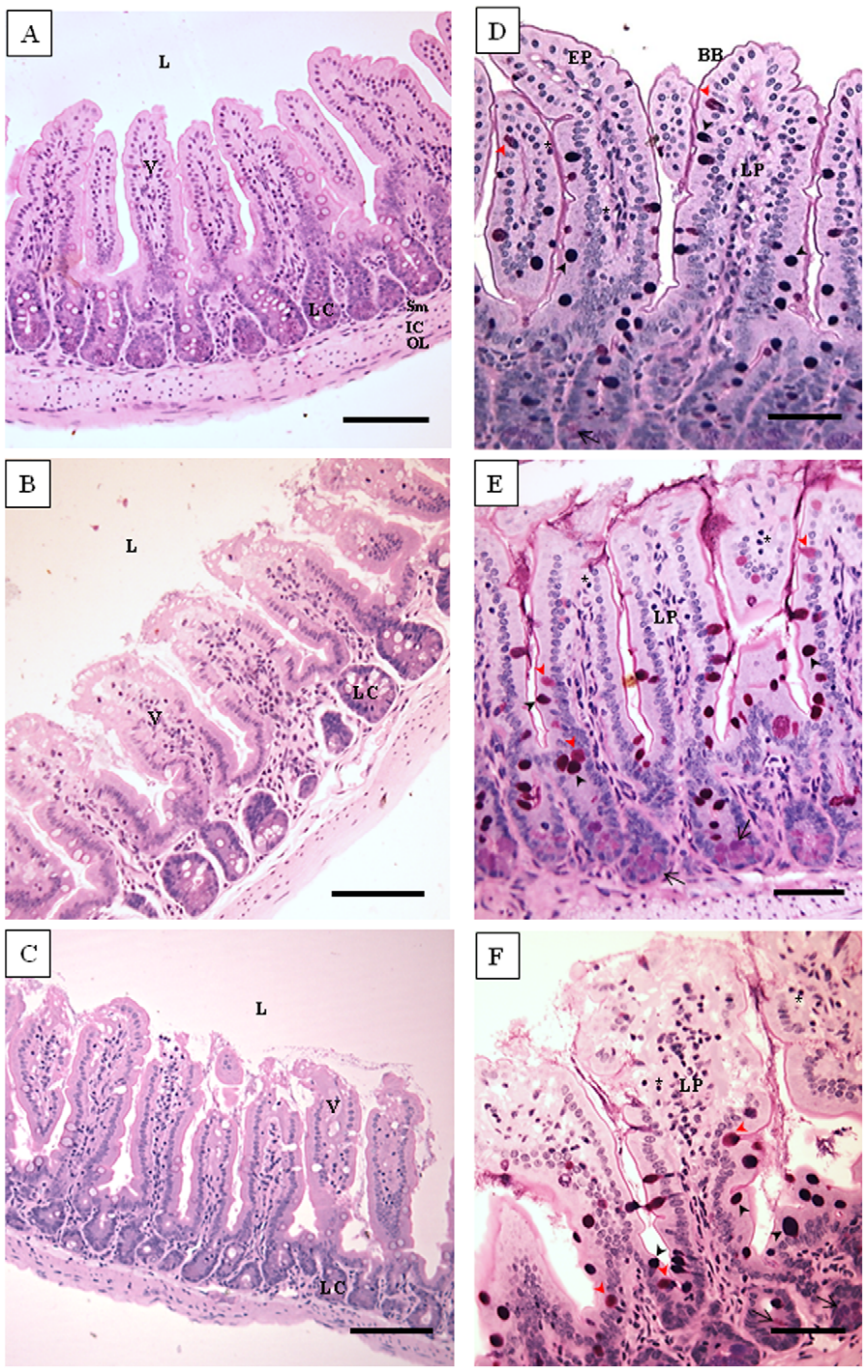
Photomicrographs of histological sections of small intestine of the animal groups studied. Jejunum segments were collected and processed for optical microscopy analysis at the end of the experiment. (NC), negative control group, figures A and D; (V) mice treated with viable *S. bovis* HC5 cells, figures B and E; (HK) mice treated with heat-killed *S. bovis* HC5 cells, figures C and F. The sections were stained with hematoxylin and eosin (HE; right panel) or PAS/Alcian Blue (left panel). Abbreviations: L: lumen; EP: simple cuboidal epithelium; BB: brush border; V: villum; LP: lamina propria; LC: Lieberkühn crypt; E: edema; V: blood vessel; Sm: submucosa; IC: inner circular muscle layer; OL: outer longitudinal muscle layer. The asterisks indicate intraepithelial lymphocytes; simple arrow indicates Paneth cells. Black arrow head indicates goblet cells PAS/AB^+^; red arrow head indicates PAS^+^ cells. Right panel – Scale bar: 100 µm; Left panel – Scale bar: 50 µm.

The small intestine of the NC group presented well-preserved villi and crypts with intact intestinal layers (Figure 4A). In the V group, histological analysis indicated the presence of eosinophils in the lamina propria, development of small areas with degenerative alterations of the epithelium and reduction of the brush border (Figure 4B). In the HK group, epithelial degenerative changes, reduction of the brush border, immune cell infiltration, moderate edema and congestion of the lamina propria were observed (Figure 4C). In general, similar histological characteristics of the small intestine were observed for all animals (N = 8) within each experimental group (NC, V and HK).

**Figure 5:**
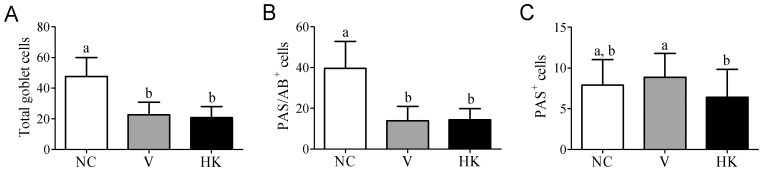
Comparison of the number of total goblet cells and mucin production among the experimental groups. (A) total number of cells; (B) PAS/AB^+^ cells; (C) PAS^+^ cells. Data were shown as average±SEM. Differences among treatments were indicated by different lowercase letters and were considered statistically significant by the Dunn's multiple comparison test (p<0.05). (NC) negative control group; (V) mice treated with viable *S. bovis* HC5 cells; (HK) mice treated with heat-killed *S. bovis* HC5 cells (HK).

Proliferative changes in goblet cells and mucin hypersecretion are one of the main characteristics of the ongoing bacterial inflammation in mucosa. Aimed to examine the influence of *S. bovis* HC5 on small intestine goblet cells, the number and the composition of their mucopolysaccharides were compared among the groups. The number of total goblet cells present in small intestine of the mice treated with *S. bovis* HC5 (viable or heat-killed cells) was reduced and statistically different when compared to the number of cells encountered in the small intestine of the NC group (p<0.05) (Figures 4D–F and 5A).

The majority of goblet cells present in animals of the NC group were PAS/AB^+^ cells, which secrete both neutral and acidic mucopolysaccharides (83% of the total number of goblet cells). The PAS/AB^+^ cells were significantly reduced (p<0.05) in V and HK groups (61.25% and 69.44% reduction, respectively) (Figure 5B). PAS^+^ cells, which secrete only neutral mucopolysaccharides, represented a small fraction of the total number of goblet cells among the experimental groups and there was no difference in the number of PAS^+^ cells compared to the negative control group. Nonetheless, a significant difference (p<0.05) was still observed between the BALB/c mice that received viable *S. bovis* HC5 and the animals treated with heat-killed cells (Figure 5C). Cells secreting exclusively acid mucins (AB^+^ cells) were not detected in this study.

**Figure 6:**
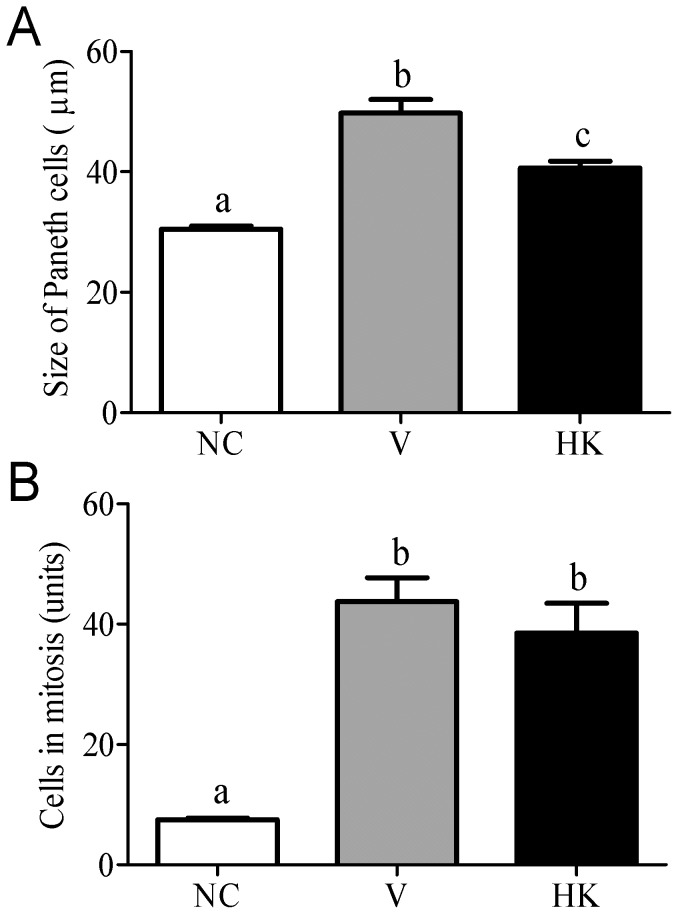
Size of Paneth cells (A) and number of cells in mitosis (B) at the small intestinal crypts of animals from different experimental groups. Data were shown as average±SEM. Differences among treatments were indicated by different lowercase letters and were considered statistically significant by the Dunn's multiple comparison test (p<0.05). (NC) negative control group; (V) mice treated with viable *S. bovis* HC5 cells; (HK) mice treated with heat-killed *S. bovis* HC5 cells (HK).

Besides the mucus production, the Paneth cells also contribute to the maintenance of the gastrointestinal barrier, preventing the access of bacteria and viruses to the underlying mucosa, by secreting antimicrobial molecules into the lumen of the intestinal crypt, when exposed to bacteria or bacterial antigens. Upon administration of *S. bovis* HC5, a significant hypertrophy of Paneth cells was observed, and this increase was more pronounced in V group (Figure 6A).

The process of epithelial renewal was highly visible in the small intestines of the animals treated with *S. bovis* HC5, and thus the presence of epithelial cells in mitotic division, which is considered an important factor in this process, was analyzed. A 4-fold increase in the number of cells in mitosis was observed in the V and HK groups, indicating a significant cell proliferation compared to the NC (negative control) group (p<0.05) (Figure 6B).

**Figure 7:**
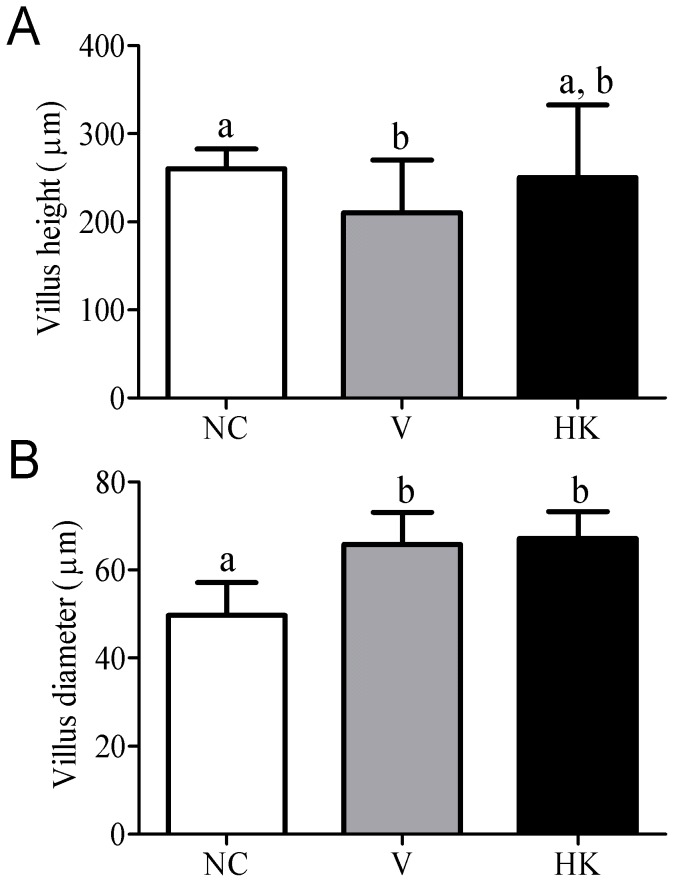
Height (A) and diameter (B) of the small intestinal villi of animals from different experimental groups. Data were shown as average±SEM. Differences among treatments were indicated by different lowercase letters and were considered statistically significant by the Dunn's multiple comparison test (p<0.05). (NC) negative control group; (V) mice treated with viable *S. bovis* HC5 cells; (HK) mice treated with heat-killed *S. bovis* HC5 cells (HK).

Changes in the architecture of the jejunum villi were evidenced when the animals were treated with *S. bovis* HC5. A significant decrease in villous height was detected only in the V group (Figure 7A), and a significant increase in the diameter of the villi was observed for the V and HK groups (Figure 7B).

**Figure 8:**
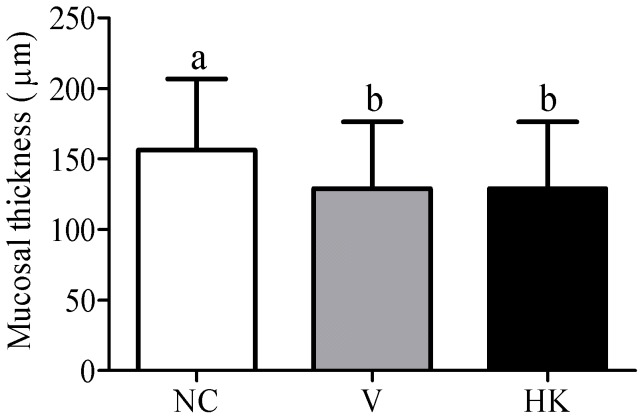
Mucosal thickness of the large intestine of the mice at the experimental groups. Data were shown as average±SEM. Differences among treatments were indicated by different lowercase letters and were considered statistically significant by the Dunn's multiple comparison test (p<0.05). (NC) negative control group; (V) mice treated with viable *S. bovis* HC5 cells; (HK) mice treated with heat-killed *S. bovis* HC5 cells (HK).

The epithelium and cellularity of the large intestine were not affected by the administration of *S. bovis* HC5 cells, but a moderate edema in the lamina propria was observed for both V and HK groups (data not shown). A significant reduction at the mucosal thickness in the large intestine was also observed among the animals treated with *S. bovis* HC5 (Figure 8).

### The viability of *S. bovis* HC5 cells is a factor that affects the pattern of cytokines produced in small intestine of BALB/c mice

**Figure 9:**
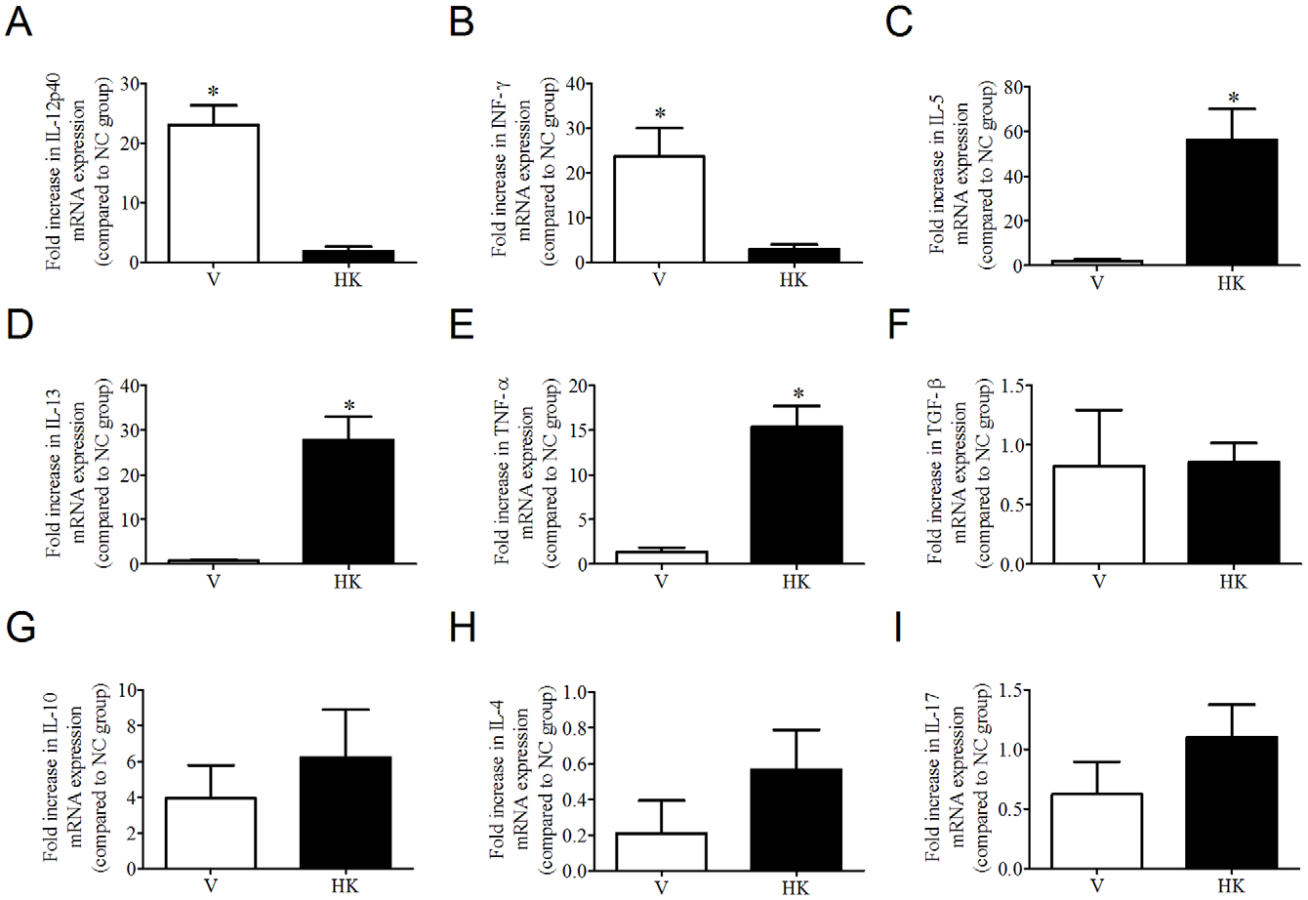
Cytokine production in small intestine of five-week old female BALB/c mice that received *S. bovis* HC5 cells. Segments of jejunum were collected on day 58 of the experiment and mRNA was extracted. The relative expression of the interleukin genes determined by real time-PCR was calculated in reference to the β-actin in each sample. (A) IL-12p40; (B) INF-γ; (C) IL-5; (D) IL-13; (E) TNF-α; (F) TGF-β; (G) IL-10; (H) IL-4; (I) IL-17. Results are shown as the mean value±SEM of duplicate samples from three independent mice within the V and HK groups, relative to the NC group. Differences between the relative cytokine expression in small intestine from mice treated with viable *S. bovis* HC5 cells (V) and treated with heat-killed *S. bovis* HC5 cells (HK) were indicated by (*) and were considered statistically significant by the t test (p<0.05).

In order to investigate the effects of *S. bovis* HC5-oral administration to the host immune system *in vivo*, we used real time RT-PCR to evaluate the patterns of relative expression of cytokine genes in spleen and small intestine (Figure 9). Comparing the groups that received *S. bovis* HC5 cells, the IL-12 and IFN-γ mRNA expression was significantly higher in the small intestine of the mice belonging to the V group (p<0.05, Figures 9A and 9B), while the relative expression of IL-5, IL-13 and TNF-α was significantly increased in the small intestine of HK group (Figures 9C, 9D and 9E). The mRNA levels of the regulatory cytokines TGF-β and IL-10 were basically the same for V and HK groups, and the expression of IL-4 and IL-17 also did not differ between the groups (Figures 9F–I).

No differences in cytokine mRNA expression were found in the spleen of animals receiving the V and HK cells (data not shown).

## Discussion

The use of direct fed microbials (DFM) in livestock is not new. However, there is a lack of information regarding the safety of bacteriocin-producing bacteria during passage through the GI tract of ruminant and monogastric animals. In this regard, it is of special interest to study the histological alterations and immunostimulatory response induced by orally administered microbes. Previous *in vivo* studies indicated that viable and non-viable *Lactobacillus plantarum* cells showed a similar cytokine production when orally administered to mice [33]. Nonetheless, humoral immune response appears to vary when animal models are exposed to viable and non-viable *Streptococcus* cells [26], [34]. No studies so far have addressed the effects of the administration of the bacteriocin-producing bacterium *Streptococcus bovis* HC5 to an animal host. In the present study, we determined the local and systemic effects of the oral administration of *S. bovis* HC5 to pre-sensitized BALB/c mice. Viable and heat-killed cells of *S. bovis* HC5 were used in order to determine the differences in response induced by these cells in our *in vivo* animal model.

The treatment with *S. bovis* HC5 has not impaired the weight gain of BALB/c mice, but the animal gastrointestinal physiology was influenced by the viability of *S. bovis* HC5 cultures. Upon oral challenge with heat-killed *S. bovis* HC5 cells, the gut permeability to proteins was indeed altered, as evidenced by greater uptake of the bystander protein β-LG, when compared to the negative control group. In the V group, some β-LG was detected after 0.5 h of administration, but the β-LG passage was still 50% lower compared to the animals of the HK group.

Balzan *et al.* [35] demonstrated that greater intestinal permeability can increase the risk of bacterial translocation. However, hyperpermeability is not a determinant factor for bacterial translocation. Bacterial translocation was initially defined as the passage of viable intestinal bacteria through the gut mucosa into normal sterile tissue [36]. This concept was expanded to include the translocation of endotoxins or antigens from the intestinal lumen into the circulation, causing systemic inflammation and distant organ injury [35]. Bacteria translocate via intestinal epithelial cells (transcellular route) or by using the tight junctions (paracellular route) [35]. In healthy hosts, with an intact intestinal barrier and a competent immune system, indigenous bacteria “spontaneously” translocate across the intestinal epithelial barrier at a low rate, which is considered a physiologic event. In this situation, the majority of bacteria that translocate are destroyed by innate immune defenses of the host, without deleterious consequences [37].

Although bacterial translocation to the mesenteric lymph nodes were not determined in our *in vivo* assays, which would be considered a direct evidence of bacterial translocation [38], extensive histological analysis did not reveal the passage of bacterial cells from the gastrointestinal lumen to the lamina propria or other tissues. The rate of translocation varies among bacterial species, and previous work demonstrated that facultative anaerobic *Enterobacteriaceae* translocate most readily from the GI tract [39]. The Gram-positive, oxygen-tolerant bacteria, such as *Staphylococcus epidermidis*, translocate at an intermediate rate, while strict anaerobes, such as *Bacteroides*, *Clostridium*, and *Fusobacterium*, translocate at the lowest rate, even though they normally occur at very high levels in the GI tract (10^10−11^/g caecum). In addition, evidences suggest that oxygen sensitivity may be a limiting factor in the translocation of anaerobic bacteria [40].

In the present work, the sensitization of the animals probably triggered the immune system to respond to *S. bovis* HC5 antigens, with the majority of bacterial cells being phagocytosed. This activity may contribute to the intestinal inflammatory response and prevent bacterial translocation. This idea is supported by the absence of histological alterations in the extra-intestinal sites (e.g. spleen, liver and heart) of the animals treated with *S. bovis* HC5.

The significant reduction on the total number of splenocytes observed when the animals were treated with *S. bovis* HC5 cells may be due to the migration of immune cells to the intestine, [41], [42], where the main histological alterations were observed. Edema and congestion in the small intestine were often similar for both V and HK groups. The administration of *S. bovis* HC5 caused the flattening of the villi in the small intestine, but a more prominent reduction of the villous diameter was detected among the animals of the V group. These alterations were probably caused by the direct effect of the bacteria or their components on the small intestinal epithelial cells. Similar results were obtained when viable cells of *Lactobacillus delbrueckii* UFV-H2b20, a probiotic lactic acid bacterium, were administrated to mice [43].

In general, the presence of substances in the gastrointestinal tract influence the expression and activity of key proteins involved in the regulation of cell proliferation, differentiation and apoptosis [44], [45]. In both V and HK groups, a hypertrophy of Paneth cells and also an increase in mitotic activity were observed, indicating that despite the loss of villi architecture, the processes of antimicrobial compounds secretion, cell turnover and tissue repair remained activated, in an attempt to counteract the injuries caused by *S. bovis* HC5 cells in the intestine. Again, the effects on the Paneth cells and mitotic division were more prominent in the mice that received viable cells.

The number and the secretory activity pattern of goblet cells can be affected by a variety of stimuli, including the exposure to bacteria, alterations of the normal microbiota and inflammatory mediators [46]. It has been suggested that acidic mucins are less degraded by bacterial glycosidases and host proteases, thus protecting against bacterial translocation [47]. In this sense, an increase in acid mucin producer goblet cells (AB^+^ cells) were expected in response to the exposure to *S. bovis* HC5. However, cells secreting exclusively acid mucins were not observed in this study. In addition, a reduction in the number of PAS/AB^+^ goblet cells (producing neutral and acid mucins) was observed following the oral administration of *S. bovis* HC5 cells to BALB/c mice. This effect cannot be fully explained by the reduction of mucus secretion, but could have been affected by the limited number of counting fields, which was caused by partially destructed villi in the small intestine of animals from the V and HK groups.

In contrast with the effects observed for the small intestine, animals that received viable or heat killed cells of *S. bovis* HC5 did not show epithelium degeneration and congestion in the large intestine. However, edema and mucosal atrophy were detected in the large intestine of the animals and did not differ between the V and HK groups. In the large intestine, the passage rate and physical-chemical conditions favor bacterial colonization, with a dominance of anaerobic bacteria (10^11^–10^13^ CFU ml^−1^) [48]. This dense microbial community and the production of antimicrobial substances by resident bacteria can limit the growth and colonization of alloctone bacteria arriving in the large intestine. Additionally, the microbial populations in the large intestine can influence the proliferation and differentiation of epithelial intestinal cells and also contribute to the maintenance of the mucosal immune system [49], [50]. Based on these observations, it appears that orally administered *S. bovis* HC5 cells will be limited in their ability to cause epithelial degenerative changes in the large intestine compared to the small intestine.

Despite of similar histological and morphometric alterations caused by viable and heat-killed *S. bovis* HC5 cells to the GI tract of BALB/c mice, a reduced epithelial barrier function was observed only for the HK group, as demonstrated by the increased β-LG concentration in the sera of animals receiving HK cells. Based on the degenerative epithelial alterations observed among the animals of the V group, a reduced barrier function caused by the administration of viable *S. bovis* HC5 cells could be expected. However, an increase in intestinal permeability was not observed in the V group, which could be explained by a different pattern of intestinal cytokine expression between the V and HK groups. The proinflammatory cytokines TNF-α and IFN-γ appear to induce a pathological opening of the intestinal tight junction (TJ) barrier, a mechanism that contributes to intestinal inflammation [51]. In this study, the TNF-α expression was increased in the HK group, while the levels of IFN-γ were higher in the V group. Additionally, the administration of heat killed cells induced T-helper type 2 cytokines (IL-5 and IL-13), which are also related to the increasing of intestinal permeability [52].

Some LAB can affect the host's systemic and mucosal immunity [53], [54], although the intensity and characteristics of these immune-stimulatory activities can vary among species and even among strains, being affected by the ecological niche of the microorganism under evaluation, the growth phase of the bacterium, the animal model adopted, and the doses administrated [54], [55], [56], [57], [58]. Moreover, the autolysis of bacterial cells [57], the structural modification or degradation of the effective components from bacterial cells affect the immune-stimulatory effects induced by bacteria, and have been described as an important issue for recognition by the phagocytes [59].

To determine if the events triggered by chronic exposure to viable and heat-killed *S. bovis* HC5 cells were restricted to the intestinal mucosa or whether they were part of a systemic response, the expression of cytokines was analyzed in the intestine and spleen of the BALB/c mice. In our study, systemic immune-mediated effects were not been detected by the administration of *S. bovis* HC5 cells to BALB/c mice, but local immune-mediated effects could be observed on the jejunum of these animals, and, in this case, the immune response was affected differentially by the viability of the *S. bovis* HC5 cells. We have shown that viable cells of *S. bovis* HC5 were a potent inducer of the T_H_1-type cytokines IL-12 and INF-γ by immune-competent cells in the intestinal mucosa, and, on the other hand, heat-killed *S. bovis* HC5 cells induced the production of the pro-inflammatory cytokine TNF-α, and also type 2 cytokine, IL-5 and IL-13.

Certain lactic acid bacteria induce the production of T_H_1-type cytokines, such as IL-12 and IFN-γ, and shift a T_H_2-dominant condition to a T_H_1-dominant condition [60]. In agreement with our results, the administration of the halophilic LAB *Tetragenococcus halophilus* Th221 promoted T_H_1 immunity, inducing the IL-12 production [9], and a heat-killed *Lactobacillus plantarum* L-137, a strain isolated from fermented food, was also a potent inducer of TNF-α [61]. Mohamadzadeh *et al.* [62] has also shown the effectiveness of viable cells of *Lactobacillus* on T_H_1 response, in which activation of human dendritic cells by *Lactobacillus* skews T cells toward T_H_1 polarization. *Lactobacillus gasseri* OLL2809 stimulated the production of IL-12 (p70) by murine splenocytes, and suppressed serum antigen-specific IgE levels via the T_H_1/T_H_2 balance [57].

However, Maeda *et al.* [33] did not detect pro-inflammatory cytokines in the serum of the C57BL/6 mice after oral administration of heat-killed *Lactobacillus plantarum* L-137. According to Pochard *et al*. [60], in the presence of both viable and non-viable bacterial cells of *Lactobacillus plantarum* NCIMB8826, *Lactococcus lactis* MG1363, *Lactococcus lactis* ATCC393 and *Lactobacillus rhamnosus* GG, an increased secretion of INF-ã and a reduction in the synthesis of IL-4 and IL-5 were observed in mononuclear cells from healthy and allergic patients. In a similar way, Kalliomaki *et al*. [63] reported that after oral supplementation with *Lactobacillus rhamnosus* GG, children with atopic dermatitis and allergy to cow's milk exhibited a transient increase in the production of INF-γ and a reduction 1in the production of IL-4.

In summary, the administration of *S. bovis* HC5 cells to pre-sensitized BALB/c mice induced major effects at local level, altering the permeability, morphometric characteristics and the pattern of cytokine production on the intestine of the animals, and the viability of the bacterial cells differentially affected some of the features evaluated. The mechanisms by which *S. bovis* HC5 interacts with intestinal cells and stimulate immune response, as well as the possible effects of this bacterium on the microbial community composition in the intestine, will be the subject of future studies.
